# White matter lesion load determines exercise-induced dopaminergic plasticity and working memory gains in aging

**DOI:** 10.1038/s41398-022-02270-9

**Published:** 2023-01-31

**Authors:** Bryn Farnsworth von Cederwald, Jarkko Johansson, Katrine Riklund, Nina Karalija, Carl-Johan Boraxbekk

**Affiliations:** 1 Umeå Center for Functional Brain Imaging (UFBI), Umeå University, Umeå, Sweden; 2 Department of Integrative Medical Biology, Umeå University, Umeå, Sweden; 3 Department of Radiation Sciences, Diagnostic Radiology, Umeå University, Umeå, Sweden; 4 Danish Research Center for Magnetic Resonance (DRCMR), Center for Functional and Diagnostic Imaging and Research, Copenhagen University Hospital, Amager and Hvidovre, Copenhagen, Denmark; 5 Institute of Sports Medicine Copenhagen (ISMC) and Department of Neurology, Copenhagen University Hospital Bispebjerg, Copenhagen, Denmark; 6 Institute for Clinical Medicine, Faculty of Medical and Health Sciences, University of Copenhagen, Copenhagen, Denmark

**Keywords:** Neuroscience, Pathogenesis

## Abstract

Age-related dopamine reductions have been suggested to contribute to maladaptive working memory (WM) function in older ages. One promising intervention approach is to increase physical activity, as this has been associated with plasticity of the striatal dopamine system and WM improvements, however with individual differences in efficacy. The present work focused on the impact of individual differences in white-matter lesion burden upon dopamine D2-like receptor (DRD2) availability and WM changes in response to a 6 months physical activity intervention. While the intervention altered striatal DRD2 availability and WM performance in individuals with no or only mild lesions (*p* < 0.05), no such effects were found in individuals with moderate-to-severe lesion severity (*p* > 0.05). Follow-up analyses revealed a similar pattern for processing speed, but not for episodic memory performance. Linear analyses further revealed that lesion volume (ml) at baseline was associated with reduced DRD2 availability (*r* = −0.41, *p* < 0.05), and level of DRD2 change (*r* = 0.40, *p* < 0.05). Taken together, this study underlines the necessity to consider cerebrovascular health in interventions with neurocognitive targets. Future work should assess whether these findings extend beyond measures of DRD2 availability and WM.

## Introduction

The relationship between physical exercise and brain maintenance in aging has been widely promoted during the last decade (e.g., [1–3]). However, the structural and functional brain benefits from increasing physical fitness vary greatly between individuals [4] and it is clear that “one size” of intervention does not fit all. The variability in outcome may stem from factors that limit neural plasticity, such as individual differences within brain health at study enrollment.

The dopamine system is particularly age sensitive, with an approximate 10% reduction of dopaminergic markers per decade across the human lifespan [5]. The age-sensitivity of the dopamine system has, in turn, been repeatedly linked to concordant age-related cognitive decline [6], particularly with regards to working memory (WM) [7, 8]. Physical exercise interventions have been shown to enhance cognitive capabilities in elderly populations [9, 10], potentially by acting upon and increasing dopamine availability [11–13]. However, results have been inconsistent [14–19], suggesting that as-of-yet unspecified mechanisms may be a source of individual differences in intervention gains [20]. One such individual difference, particularly within an older sample, is that of pre-existing cerebrovascular damage. White matter lesions are late-stage manifestations of cerebral small-vessel disease, and arise alongside other brain abnormalities, including inflammation and demyelination [21]. In older populations, lesions are almost ubiquitous, with an in‐life prevalence of over 90% for individuals aged over 65 [22, 23].

Individuals with severe lesions are at higher risk of cognitive decline and dementia [24, 25]. The presence of confluent lesions may further reduce neural and cognitive plasticity, as cognitive gains following interventions have been related to white-matter integrity and plasticity [26, 27]. Such negative effects may stem from lesions obstructing white-matter tracts, but also from the increased levels of proinflammatory cytokines and neurotoxic mediators, as well as maladaptive neurotrophin regulation, in individuals with severe lesions [28]. As evidence for lesion-associated treatment modulation, higher lesion burden has been associated with reduced motoric improvement following physical rehabilitation [29], and poor responses to antidepressant treatment in patients suffering from chronic depression [30]. Furthermore, elevated lesion burden has been associated with reduced dopamine integrity in healthy, older individuals [31, 32]. It therefore seems plausible that one route by which severe lesion manifestation may hamper plasticity is via dopaminergic attenuation.

The current study aims to explore whether white-matter lesion burden modulates the efficacy of physical exercise interventions by limiting dopaminergic plasticity and cognitive improvements. Analyses were performed within a sample from the Physical Influences on Brain in Aging (PHIBRA) study [33], in which older adults of ages 64–78 years were randomized into one of two treatment arms, and underwent either aerobic training or stretching and toning exercises, three times a week, for a total of six months. Participants also underwent magnetic resonance imaging (MRI) and positron emission tomography with computed tomography (PET/CT) scans, using the dopamine D2-like receptor (DRD2) antagonist ^11^C-raclopride, immediately prior and subsequent to the intervention period. We hypothesized that (i) striatal DRD2 availability is reduced as a function of lesion burden at study entrance, (ii) that individual differences in the extent of lesion-related damage would modulate intervention-related gains in striatal DRD2 plasticity and WM, and (iii) that striatal DRD2 change would be associated with WM improvements when accounting for lesion burden.

## Subjects and methods

The study was approved by the Swedish Ethical Review Authority (Umeå, Sweden; registration number: 2013-238-31M) and was carried out in accordance with The Code of Ethics of the World Medical Association (Declaration of Helsinki). Written informed consent was obtained from all participants prior to any testing.

### Sample

The analyses were performed using the participants randomized by computer to an intervention and an active control group of the PHIBRA study (*n* = 30 per group), a 6-month aerobic exercise intervention [33]. One participant from the active control group dropped out before the follow-up, and one did not have a T1-weighted image from MRI. Thus, the effective sample was 30 for the aerobic exercise intervention group, and 28 for the active control group. Between test sessions, individuals assigned to the aerobic group engaged in walking, jogging, cycling, or using cross-trainers to improve aerobic capacity, whereas the active control group performed stretching and toning to improve muscle strength, flexibility, and balance. The training was completed three times a week in separate group sessions, with individualized instruction aimed at maximizing VO_2_ improvements. Notably, both groups increased significantly in aerobic fitness over the study period [33], and were comparable for lesions volume and various cardiovascular risk factors (Supplementary Table 1). In the present work, the whole sample (regardless of intervention type) was divided according to lesion severity grade (see descriptives in Table 1). Participants underwent brain imaging with MRI, and PET/CT scans with ^11^C-raclopride, at two occasions separated by approximately 7 months. Exclusion criteria included engagement in regular physical activity, neurological and psychiatric disorders, previous head trauma, diabetes, and medications that can affect brain and cognitive functioning. Further exclusion criteria consisted of a Mini Mental State Examination (MMSE) score <27; and MRI-incompatible factors. All structural MRI images were screened for structural abnormalities by a neuroradiologist.

**Table 1:** Descriptives for mild- and moderate-to-high lesion severity groups, as dichotomized by grades 0–1 and 2–3 according to the Fazekas lesion grading scale.

	Mild lesion severity (Fazekas grade 0 or 1)	Moderate- to-high lesions severity (Fazekas grade 2 or 3)
N	35	23
Aerobic intervention vs. control group (%)	63% vs. 37%	35% vs. 65%*
Age	68.9 ± 2.5	68.7 ± 3.2
Sex	46% men	44% men
Education	14.3 ± 3.8	12.6 ± 4.5
MMSE	29.1 ± 1.0	29.3 ± 0.9
Total lesion volume (ml)	1.5 ± 1.5	7.1 ± 5.3*
CVD risk profiles (%)	23.0 ± 9.7	22.3 ± 9.9
Systolic blood pressure	143.8 ± 20.2	148.9 ± 13.6
Diastolic blood pressure	81.0 ± 9.2	85.9 ± 7.2*
BMI	26.3 ± 3.5	26.4 ± 3.2
Fat (%)	36.7 ± 6.9	37.3 ± 8.3
VO_2_ peak	21.4 ± 3.6	19.6 ± 4.0
VO_2_ peak change	5.2 ± 3.9	5.2 ± 3.3

Significant group differences *(p* < 0.05) are shown with an asterisk.

### Brain imaging: acquisition and analyses

The same scanners and protocols were used at both data collection waves (baseline and follow up). MRI was performed with a 3 tesla Discovery MR 750 scanner (General Electric, WI, US), equipped with a 32-channel phased-array head coil. PET data were acquired with a Discovery PET/CT 690 (General Electric, WI, US) and 250 MBq ^11^C-raclopride. At each data collection wave, MRI and PET was performed on two different days, separated by a maximum of 1 week.

#### Regional volumes and DRD2 availability

T1-weighted images were obtained with echo time 3.2 ms, flip angle 12°, repetition time 8.19 ms, 176 slices with thickness 1.0 mm, field of view 25.0 cm with resolution 0.98 mm upsampled to 0.49 mm. The longitudinal image processing pipeline in FreeSurfer, version 6.0 was used to process T1-weighted images and derive volume estimates of gray and white matter. Subcortical gray matter segmentations and cortical parcellations were used to define regions-of-interest (ROIs) for assessment of DRD2 availability. Cortical parcellation was performed according to the Desikan-Killany cortical atlas to delineate ROIs [34]. ROIs consisted of anterior cingulate cortex (ACC; rostral and caudal anterior divisions), frontal cortex (superior frontal, middle frontal, and inferior frontal gyri), the striatum, and hippocampus.

A 55-min, 18-frame dynamic PET scan was acquired during rest following intravenous bolus injection of approximately 250 MBq ^11^C-raclopride. PET images were motion-corrected and co-registered with the structural T1-weighted images from the corresponding session (baseline and follow-up) using the Statistical Parametric Mapping software (SPM12). DRD2 binding potential (BP_ND_) estimates were calculated using the multilinear reference-tissue model (MRTM) on dynamic partial-volume corrected data, with cerebellar gray matter radioactivity as an indirect input function (due to negligible DRD2 expression). When employing ^11^C-raclopride-derived DRD2 measurements, negative changes following dopamine-elevating interventions are generally interpreted as increases in extracellular dopamine levels [35]. Further details are provided in the Supplementary Methods.

#### White-matter lesions

**Fig. 1:**
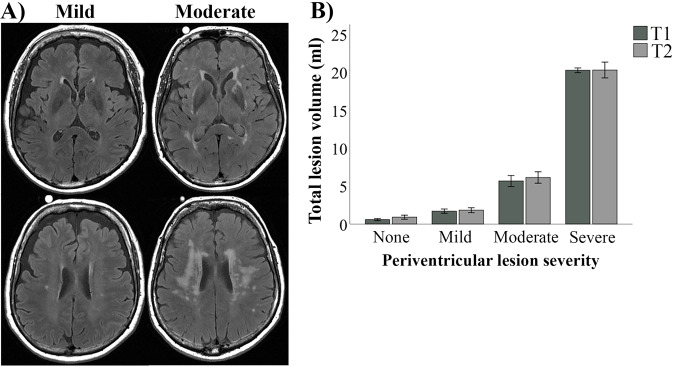
White-matter lesion severity. Large individual differences in lesion severity was observed in the sample, where the majority had mild (Fazekas grade 1), or moderate (Fazekas grade 2) periventricular lesion severity **A**. Severity grade was associated with sizeable differences in total lesion volume at the two test occasions **B**. *T1* timepoint 1 - baseline, *T2* timepoint 2 - follow-up.

Periventricular white-matter lesion severity was assessed (by author N.K.) according to the Fazekas grading scale [36]. Lesions forming a thin lining around the lateral ventricles were assigned grade 1, lesions appearing as a smooth halo with minimal confluence were assigned grade 2, and large, confluent lesions extending into the deep white matter as grade 3 (Fig. 1a). Individuals without visible lesions were graded 0. Further information regarding lesion-severity groups (grade 0–1 vs. 2–3) is provided in Table 1. Additionally, total lesion volume was automatically segmented from fluid-attenuated inversion recovery (FLAIR) images, details of which are provided in the Supplementary Methods.

### Working memory assessments

WM was assessed via three computerized tasks: letter memory, numerical n-back, and a keep-track task. The dependent variable was the sum of correct answers, multiplied by the number of target categories per block. The sum scores per task were standardized (Z score), and averaged across the three tasks to build one WM composite score per individual. To assess whether effects would transfer to other age-sensitive cognitive functions in addition to our primary dependent variable (WM), episodic memory and processing speed were considered in a second step. Details for all cognitive measures are found in the Supplementary Methods, and in previous work [33].

### Assessment of health and physical fitness

Body-mass index was calculated from height and weight, and percent of fat was measured with Dual X-ray absorptiometry (DXA). Blood pressure was measured in a sitting position. 10-year cardiovascular disease risk (%) was calculated from hypertension and diabetes diagnosis, systolic blood pressure, BMI, smoking, age, and sex, according to an established model [37].

Aerobic fitness was assessed via standardized graded cycle ergometer tests, wherein the resistance was increased by 30 W every 3 min. Starting values were 30 W for women, and 40 W for men. Expired air was measured through a mouthpiece and analysis of oxygen uptake every 20 s (VO_2_ = ml O_2_ per kg and minute). VO_2_ peak was estimated as the highest VO_2_ reached before test termination. The study protocol has previously been described in detail [33].

### Statistical analyses

Analyses were performed with SPSS (version 26), the graphical modeling software Ωnyx (http://onyx.brandmaier.de), and R [38]. Distributions for health, lifestyle, DRD2, and cognitive variables were normally distributed (skewness and kurtosis between −1 and 1). Lesion volume was skewed (skewness: 2.4 and 1.3, kurtosis: 6.5 and 2.4 at baseline and follow-up, respectively) and therefore logarithmically transformed. Remaining univariate outliers were defined as values >3.29 SD from the mean [39] at each timepoint (*n* = 2 for lesions at baseline and follow-up).

**Fig. 2:**
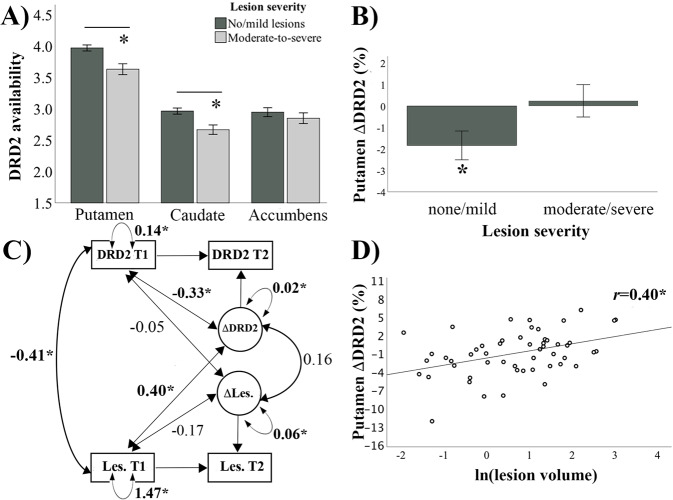
Lesion severity and putamen DRD2 availability. The group with elevated lesion severity displayed reduced DRD2 availability in putamen and caudate at baseline **A**, and lack of significant intervention-related DRD2 change for putamen **B**. Linear relationships were found between baseline lesion volume and baseline DRD2 levels, as well as for baseline lesion volume and the degree of DRD2 change **C**, **D**. *p < 0.05. *DRD2* dopamine D2-receptor, *T1* timepoint 1 - baseline, *T2* timepoint 2 - follow-up, *Les.* lesions, Δ change.

**Fig. 3:**
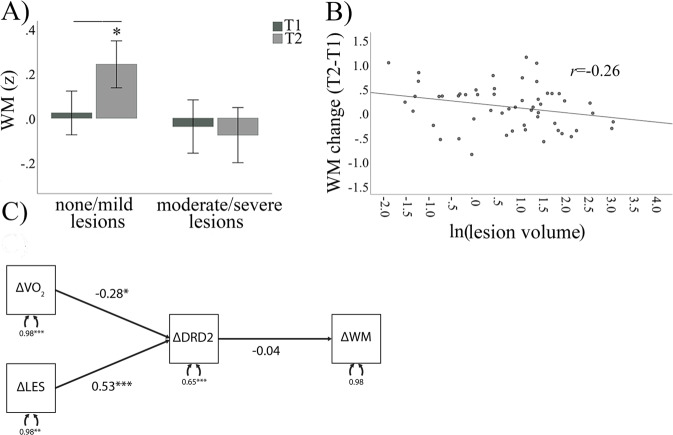
Lesion severity and working memory. Working memory gains were restricted to the group of individuals with mild lesion severity **A**, with a trend for a linear association **B**. A path analysis showed association between change in VO_2_ peak and lesion volume in relation to putamen DRD2 change **C**. No links were found between putamen DRD2 change and working memory change. Working memory is expressed as a Z-score. Each value is adjusted for age, gender, and education. *p < 0.05. *WM* working memory, *LES* lesions, *DRD2* dopamine D2-receptor, *T1* timepoint 1 - baseline, *T2* timepoint 2 - follow-up, Δ change.

Between-group comparisons at baseline were achieved via chi-squared tests for categorical variables, and t-tests for continuous variables (Table 1 and Supplementary Table 1), or analysis of univariate or multivariate variance or covariance (ANOVA or ANCOVA, the latter controlling for age, gender, and education). Change over time was assessed with repeated-measures ANOVA, ANCOVA, paired samples t-tests, and bivariate difference score models (Figs. 2 and 3). Correlations are reported with Pearson’s correlation coefficient (*r*). Furthermore, a hierarchical regression was used to assess whether lesion volume predicted DRD2 change above and beyond factors related to cardiovascular risk at baseline.

Regional DRD2 availability is presented as a mean value over left and right hemispheres. To define patterns of DRD2 baseline levels and DRD2 change following the intervention, we carried out a principal component analysis (PCA). Specifically, PCA was performed to inform us of whether intervention-related DRD2 change correlated between the principal a priori region of interest (striatum), in relation to other associative regions such as the hippocampus, frontal cortex, and anterior cingulate cortex; or whether change in striatum formed an independent component that was distinct from a global test-retest effect. The selection of regions was based on previous reports, and included regions in which DRD2 levels are age-sensitive and associated with WM performance [40–42]. Loadings above 0.5 were considered meaningful [43]. An oblique rotation method (Oblimin with Kaiser normalization) was applied, as the assumption was that DRD2 components may be correlated [44]. Components with Eigenvalues >1 in a rotated solution are reported, for which factor scores were extracted and used in subsequent analyses.

Bivariate difference-score models were set up to assess baseline-change and change-change associations for white-matter lesion volume and DRD2 availability. The obtained parameters include associations between lesions and DRD2 at baseline, change-change correlations, and standardized covariances between baseline levels in variable a and change in variable b (and vice versa). Z-values >1.96 or < −1.96 indicate statistical significance at *p* < 0.05. The models are re-expressions of simple difference scores, i.e., saturated models that always yield perfect fit to the data. Therefore, we do not report measures of goodness-of-fit.

Finally, we used path analysis to examine the associations between changes in VO_2_ peak (as a measure of physical fitness), lesion volume, DRD2 availability, and WM performance. Goodness-of-fit was evaluated by comparative fit index (CFI), the Tucker-Lewis index (TLI), and the root mean square error of approximation (RMSEA). All dependent variables (percentage changes) were regressed on age, gender and education.

## Results

### White-matter lesion severity and patterns of intervention-related D2-receptor changes

The group with aggravated lesion severity (Fazekas grading 2 and 3) was characterized by significantly elevated lesion volume (t(22) = 4.7, *p* < 0.001), and higher diastolic blood pressure (t(56) = 2.1, *p* < 0.05; Table 1). Lesion severity grade also corresponded with a step-wise increase in total lesion volume for the sample (F(1, 53) = 33.0 and 35.6 at baseline and follow-up, respectively, *p-*values < 0.001; Fig. 1b). Lesion volume increased slightly over 6 months (0.3 ± 0.7 ml; F(1, 51) = 8.4, *p* = 0.006), with no differences between exercise intervention groups (F(1, 51) = 0.9, *p* = 0.356), or as a function of lesion severity at baseline (F(1, 51) = 2.3, *p* = 0.138). Measurements of total gray matter were not found to differ across lesion severity groups (F(1, 51) = 0.3, *p* = 0.573), and were not found to significantly change over time (F(1, 107) = 0.02, *p* = 0.883). Furthermore, the distribution of lesion quantity and severity was comparable across exercise intervention groups (see Supplementary Table 1).

As previously reported, both the aerobic training group and the active control group increased in aerobic fitness over the study period, albeit the increase was significantly larger for the aerobic training group [33]. Notably, DRD2 changes pre- vs. post-intervention were comparable across exercise groups [13], and exceeded the estimated annual DRD2 decline rates [5, 40]. DRD2 levels at baseline and DRD2 changes were assessed in a data-driven manner via principal component analyses; one for baseline DRD2 levels, and one for DRD2 changes. Baseline DRD2 availability variance was primarily explained by an extrastriatal component (47%), and additionally, a striatal component (23%; model 1 in Table 2). As in previous work with ^11^C-raclopride, these striatal and extrastriatal factors were correlated (*r* = 0.35, *p* = 0.008; [44]). In the model for DRD2 change, all regions, excluding the nucleus accumbens (Nacc), loaded onto one large component for DRD2 change (52% of variance). However, change in putamen and Nacc DRD2 availability was explained by a second factor (20% of variance; model 2 in Table 2). Factor scores for the two change components were not significantly correlated (*r* = 0.23, *p* = 0.08). Region-per-region analyses showed that significant change over time was restricted to the putamen (F(55) = 5.0, *p* = 0.029), while a similar, yet non-significant trend was observed for the Nacc (F(55) = 3.0, *p* = 0.087). As significant change was only observed in the putamen, we decided to restrict further analyses to this region.

**Table 2:** Results from principal component analyses for DRD2 levels at study entrance (model 1), and for DRD2 change (model 2).

Model 1: DRD2 start	Component 1 (47% of variance)	Component 2 (23% of variance)
Putamen	0.38	0.94
Caudate	0.18	0.87
Nucleus accumbens	0.29	0.58
Hippocampus	0.73	0.44
Cingulate cortex	0.89	0.25
Frontal cortex	0.90	0.27
**Model 2: DRD2 change**	**Component 1 (52% of variance)**	**Component 2 (20% of variance)**
Putamen	0.55	0.76
Caudate	0.77	0.22
Nucleus accumbens	0.07	0.90
Hippocampus	0.70	0.06
Cingulate cortex	0.88	0.24
Frontal cortex	0.92	0.26

Components with Eigenvalues > 1 are shown. Coefficients > 0.50 are highlighted with black font color.

### White-matter lesion volume modulates D2-receptor availability and plasticity

Next, we assessed whether lesion severity at baseline was associated with baseline striatal DRD2 levels (using data from component 2 in model 1), and also, explained variance in striatal DRD2 change (using data from component 2 in model 2). We found that lesion severity was predictive of baseline DRD2 status as well as DRD2 change (F(2, 48) = 8.0, *p* = 0.001). More specifically, individuals with aggravated lesion severity (Fazekas grade 2 and 3) had lower striatal DRD2 levels at baseline, as compared to those with no or mild lesions (F(1, 49) = 14.9; *p* < 0.001). Region-wise tests revealed that aggravated lesion severity was associated with reduced DRD2 availability in putamen (F(2, 48) = 10.2; *p* < 0.001; Fig. 2a), caudate DRD2 availability (F(2, 48) = 6.2; *p* = 0.004), but not Nacc DRD2 availability (F(2, 48) = 1.1; *p* = 0.337). Furthermore, striatal DRD2 change (component 2) was restricted to individuals with no or mild lesions (F(1, 49) = 4.5, *p* = 0.039), and as revealed from region-wise analyses, was found for the putamen (F(1,49) = 5.1, *p* = 0.031; Fig. 2b), but not for the Nacc (F(1,49) = 2.0, *p* = 0.164). It should be noted that a negative change in ^11^C-raclopride BP_ND_ in intervention settings is typically interpreted as ligand displacement by elevated levels of endogenous dopamine [35]. Pharmacological interventions that are known to increase endogenous dopamine amounts have been shown to decrease ^11^C-raclopride binding [45], while interventions causing a depletion of dopamine have been shown to increase ^11^C-raclopride binding [46]. In contrast, lesion severity did not predict DRD2 levels or change in extrastriatal regions (first components in model 1 and 2 in Table 2; F(2, 48) = 1.1, *p* = 0.334).

Difference-score models confirmed linear associations between baseline lesion volume in relation to baseline DRD2 levels and DRD2 change in putamen (Fig. 2c, d). Similar correlations between baseline lesion volume and putaminal DRD2 change was observed for the intervention and active control groups (see correlations in Supplementary Fig. 1a, and bivariate difference score models in Supplementary Fig. 2). Furthermore, the 6-month DRD2 reductions within the current study were several times greater than the estimated annual DRD2 reduction in aging [40]. Thus, these likely do not represent age-related DRD2 loss, but rather result from increased dopamine following the intervention.

Subsequently, we assessed whether lesion volume predicted DRD2 change above and beyond factors related to cardiovascular risk at baseline (VO_2_ peak, cardiovascular disease risk, BMI, body fat, and systolic blood pressure). While the model with only cardiovascular risk factors was not significant (F(5, 50) = 1.2; *p* = 0.316), the addition of lesion volume explained significantly more variance (F_change_ = 11.7, *p* = 0.001). In the second model (F(6, 50) = 3.2, *p* = 0.011), lesion volume (t = 3.4, *p* = 0.001) and BMI (t = −2.6, *p* = 0.012) were significantly associated with putamen DRD2 change.

### Exercise-related WM gains are restricted to individuals with mild lesion severity

There were no WM differences as a function of lesion severity at baseline (F(1, 48) = 0.2, *p* = 0.663), nor across exercise intervention groups (F(1, 48) = 0.8, *p* = 0.390). However, significant differences in WM changes were found across lesion severity groups post-intervention (F(1, 50) = 4.9, *p* = 0.032). Specifically, no WM gains were found for individuals with aggravated lesion severity (t(22) = 0.5, *p* = 0.661), whereas the group with no or mild lesions showed significant WM improvements (t(33) = 2.8, *p* = 0.008; Fig. 3a). Accordingly, a non-significant trend for a negative association was found between baseline lesion amount (ml) and WM changes (F(1, 48) = 3.0, *p* = 0.091; Fig. 3b). When assessing these association separately in the intervention and active control group, respectively; we found a significant association in the active control group (*r* = −0.48, *p* < 0.05; Supplementary Fig. 1b), but not in the intervention group (*r* = −0.02, *p* > 0.05). Secondly, we assessed whether similar effects are found for other age-sensitive cognitive measures. A similar trend was found for processing speed as for WM, where individuals with low, but not high, lesion severity grade tend to improve over time (F(1,50) = 3.9, *p* = 0.054; see Supplementary Fig. 3). Episodic memory change was, however, comparable for lesion severity groups (F(1,50) = 0.05, *p* = 0.833).

### D2-receptor availability and working memory

Path analysis was used to test associations between percentage changes of VO_2_ peak, lesion volume, putamen DRD2 BP_ND_, and WM scores (Fig. 3c). The model shows a negative association between change in VO_2_ peak and DRD2 change in putamen, suggesting that intervention-based increases in physical fitness resulted in reduced ^11^C-raclopride binding, possibly via competitive displacement from elevated levels of endogenous dopamine [35]. Accordingly, the model also shows a linear relationship between change in lesion volume and change in putamen DRD2 availability, suggesting that a relatively lower lesion volume is associated with intervention-based reductions in ^11^C-raclopride binding as a result of competitive dopamine displacement. Contrary to our third hypothesis, the model does not however show a significant relationship between changes in putamen DRD2 availability and WM performance. The model fit was acceptable, with a CFI of 0.995, a TLI of 0.990, and an RMSEA of 0.024 [47, 48].

## Discussion

The present study tested the influence of white-matter lesion load on dopaminergic and cognitive plasticity. The results support earlier observations of associations between compromised striatal DRD2 integrity and increased lesion burden in aging [31, 32]. We further show that DRD2 plasticity as well as WM improvements are limited to participants without, or with minimal lesions at study start. Periventricular lesions are located in e.g., the cingulum and corpus callosum, and disrupt the integrity of these white-matter tracts. Negative effects of ischemia, inflammation, and oxidative stress may, via proximity, extend to the striatum. Furthermore, the integrity of the cingulum and corpus callosum has been found central for WM performance [49]. We therefore suggest that the extent of lesion severity can be an influential determinant of physical exercise intervention effects on brain health. Notably, both the mild and the moderate/severe lesion group showed similar improvement of cardiovascular fitness, hence it is not the case that high lesion load limits the training effects, but rather, it limits the potential for inducing neuroplastic changes. A similar pattern was found for changes in processing speed, however, more research is needed to assess whether the scope of such influence extend to cognitive domains and brain measures beyond DRD2 plasticity and WM improvements. Interventions that aim at promoting beneficial brain aging could therefore benefit from considering lesion load at study entrance when selecting a cohort and evaluating intervention success.

Physically active individuals are generally considered to be protected from unsuccessful neurocognitive aging [1, 50]. While it is well-acknowledged that the benefits from health-promoting interventions may vary across subjects due to differences in health or genetic factors, no study has as-of-yet investigated the impact of brain health. White-matter lesions are a common finding in the aging brain, and may be predictive of future stroke, cognitive decline, and dementia [25]. Lesions have been linked to accelerated biological aging already in midlife [51]. As such, lesion manifestation may constitute a critical determinant of individual aging trajectories. The present work shows small but significant increases in lesion volume over six months, with no difference between groups engaged in different levels of exercise intensity. While increased exercise may have slight effects on white matter plasticity [52], previous work has largely shown null effects of physical exercise intervention on lesion progression [53–55]. The lesion areas are characterized by neuroinflammation and oxidative stress, thus viable preventive approaches may instead consist of anti-inflammatory treatments [56].

The current study is the first to examine the impact of lesion load upon in vivo dopaminergic plasticity. As such, it offers a framework by which to explain the discrepancy in results regarding dopaminergic change with human participants in physical exercise interventions. Studies of dopamine or dopamine-ß-hydroxylase presence in the blood found an increase [15], no change [14], or mixed findings dependent on the exercise type [16]. A more recent study, analyzing the data also used in the current study, found a relationship between striatal DRD2 availability at baseline and aerobic fitness in older adults, yet no difference in DRD2 availability in either exercise group following the intervention [13]. Physical activity intensity, rather than frequency, has also been linked to DRD2 availability [17]. Furthermore, the availability of DRD2s and dopamine transporters was found to increase following exercise interventions in groups of patients with methamphetamine-dependence or Parkinson’s disease, respectively [18, 19]. The current study provides a potential rationale for discrepancies across previous studies, by highlighting the impact that white-matter lesion load can impart upon dopamine regulation in combination with physical exercise. As is seen in the path analysis in Fig. 3c, lesion volume and change in cardiovascular fitness were both significant modulators of striatal DRD2 change across both exercise groups, indicating that the form of exercise may not be critical, as long as it impacts cardiovascular fitness positively and the subject is devoid of cerebrovascular damage.

The mechanisms by which white-matter lesions impair brain plasticity remain inconclusive, but have been suggested to encompass impaired neurogenesis and neuronal repair, elevated levels of pro-inflammatory cytokines, and dysfunctional neurotrophin regulation [28, 57, 58]. The selective link between lesions and *striatal* DRD2 availability may be explained by the increased susceptibility to ischemic events in the lenticulostriate arteries [59]. Accordingly, reductions are found for striatal pre- and postsynaptic dopaminergic markers as a function of increasing lesion burden [32, 60]. That said, there are indications of that lesion progression, over longer durations, may also impair extrastriatal DRD2 integrity, e.g., in the hippocampus [31]. The presence of lesions may thus hinder the physiological mechanisms that must take place in order for behavioral improvements to occur. Serving as an example of this, a recent study showed that motor learning following physical exercise was dependent on changes in frontal cerebral blood flow as well as white-matter plasticity [61]. In the case of WM, improvements have previously been found to be mediated by dopaminergic changes [8], and increased DRD2 signaling is critical for improved learning following physical exercise [62]. Consequently, it seems plausible that white-matter damage limits dopaminergic plasticity, and thereby, WM improvements.

The VO_2_-DRD2 link in the path analysis supports that DRD2 reductions follows improved physical fitness, presumably via increased endogenous dopamine, causing competitive displacement of ^11^C-raclopride [45, 46]. Possible mechanisms by which dopamine levels are altered in response to VO_2_-changes may encompass altered dopamine synthesis capacity (via aromatic L-amino acid decarboxylase), dopamine storage (via the vesicular monoamine transporter), dopamine metabolism (via catechol-O-methyltransferase and monoamine oxidase), and dopamine re-uptake (via dopamine transporter availability). Nonetheless, the hypothesis that aerobic fitness would, via increase physical fitness [63–65], give rise to WM improvements via DRD2 changes was not supported. Reasons for this may be that the DRD2-WM links are not necessarily direct nor linear [42, 66], or that additional factors need to be considered, e.g., brain structure and function, health, lifestyle, and genetic factors. To exemplify, one polymorphism in the DRD2 gene may influence ligand binding, and thereby, the DRD2-cognition link [67]. Furthermore, the lesion-WM link being restricted to the active control group, underlines that the link between lesion burden and dopaminergic plasticity is clearer than the link to behavior. One important limitation is that the path analysis indicated no significant variance in WM change, which limits interpretations of brain-WM change associations. That is, without significant individual difference in change, associations among changes cannot be meaningfully addressed. Hence, additional studies are needed to further understand the dopamine-cognition link, and whether it can be modulated in aging.

Taken together, our findings suggest that interventions aimed at improving WM and dopaminergic plasticity in older healthy individuals should consider screening for lesions before implementation. A central strength of the PHIBRA study design is its longitudinal format, the assessment of multiple indicators of the intervention gains (aerobic fitness, brain integrity, cognition), and a sample size that is above a typical in vivo dopamine study (mean *n* < 30). Still, further research is needed to explore (i) whether the findings extend to other cognitive domains and brain measures aside from WM and DRD2, (ii) how to slow the formation of lesions to facilitate continual brain plasticity into older ages, and (iii) the implications of dopaminergic plasticity for cognition in aging [5].

## Supplementary information

Supplementary material

## Data Availability

The anonymized data sets analyzed in this study is available from the corresponding author upon reasonable request from a qualified investigator. Prerequisites encompass approval of a formal project outline, a data sharing agreement, and ethical permission for the outlined research questions.
