# Supplementary material for: White matter lesion load determines exercise-induced dopaminergic plasticity and working memory gains in aging
Source: Transl Psychiatry. 2023 Jan 31;13:28. doi: 10.1038/s41398-022-02270-9 (PMC9889313; doi:10.1038/s41398-022-02270-9)
Supplement: Supplementary file 1 — Supplementary material [file 41398_2022_2270_MOESM1_ESM.docx]

**Supplementary methods:** Includes details of DRD2 availability measurements, working memory assessments, and automatic segmentation of white matter lesion burden.

**Supplementary methods**

DRD2 availability

Head movements during the PET sessions were minimized by attaching individually fitted masks to the bed surface. An attenuation CT scan (20 mA, 120 kV, 0.8 s/revolution) preceded tracer injection. Attenuation- and decay-corrected images (47 slices, field of view = 25 cm, 256 × 256-pixel transaxial images, voxel size = 0.977 × 0.977 × 3.27 mm3) were reconstructed with the iterative algorithm VUE Point HD-SharpIR (GE; 6 iterations, 24 subsets, 3.0 mm post filtering), yielding full width at half maximum (FWHM) of 3.2 mm.

Regional PVE correction was conducted using the symmetric geometric-transfer matrix implemented in FreeSurfer (1). The PVE-correction approach included (i) initial correction via resolution modelling in the iterative image reconstruction procedure (SHARP-IR), and (ii) remnant PVE using the ROI-based geometric transfer matrix approach. The size of the secondary correction kernel was estimated empirically (point-spread function (PSF) of 2.5 mm; isotropic, FWHM), to achieve a similar level of correction as in earlier dopamine studies (2). The FreeSurfer segmentations and pre-processed PET data were used to estimate PVE-corrected regional radioactivity concentrations in each ROI and time frame.

Working memory assessments

In letter memory, a sequence of the letters A, B, C, or D was randomly displayed (2 seconds per letter; inter-stimulus interval (ISI): 1 second). The task was to indicate the last four letters in the array by pressing the corresponding key. Two practice blocks preceded 8 task blocks. In the numerical n-back task, participants were to indicate with a key press whether the current digit on screen was the same digit as the digit presented 1 stimulus (1-back) or 2 stimuli (2-back) prior (20-digit sequence; 1.5 s per digit, ISI: 500 ms). A practice 1- and 2-back block preceded two 1-back blocks, and four 2-back blocks, where accuracy in the latter was the dependent variable. In the keep-track task, 15-word sequences were presented (1.5 s per word, ISI: 500 ms). Each word belonged to six categories (colors, countries, animals, etc). In each test block, words for 2-4 target categories were presented in boxes, and the task was to recall the last words belonging to each target category. Two practice blocks preceded six task blocks, each with three or four target variables.

Episodic memory and processing speed assessments

Episodic memory was assessed with three tasks: word recognition, free recall, and a paired associates task. For word recognition, 30 nouns were studied (3 seconds per word, ISI: 1 s). 25 minutes later 15 words from the list and 15 novel words were presented (4 second per word, ISI: 1 s) and participants were to indicate whether they had seen the word during the encoding phase. A practice block of 5 words preceded the task blocks. In free recall, 16 nouns were presented (3 seconds per word, ISI: 1 s). Following presentation of nouns, participants were to write down as many of the words as possible. In the paired-associates task, 10 word-pairs were presented (3 seconds per word pair, ISI: 500 ms). This was repeated over two blocks. Following, participants were presented with one of the words, and were asked to write down the missing word. One practice trial of 5 word-pairs preceded the task blocks. Accuracy was the dependent measure for all three episodic memory tasks.

Processing speed was evaluated via three tasks: trail-making, digit-symbol coding, and letter comparison. During the trail-making tasks, participants connected numbers 1-16 and letters A-P (with pen and paper), respectively, as quickly as possible. Lines were not allowed to cross. The outcome variable was the total time (s) to finish the two tests. Digit-symbol coding was assessed via a computerized version of the WAIS digit-symbol coding task. Participants were instructed to indicate with a key press whether a digit-symbol combination could be found in an array with nine digit-symbol coding combinations. One practice block (10 items) preceded three task blocks of 30 trials each. In letter comparison, two letters were presented simultaneously, and participants were to indicate, as fast as possible, whether letters were identical or not. One practice block (10 items) preceded two task blocks of 30 trials each. The outcome measure was reaction time for both digit-symbol coding and letter comparison.

Each task was z-transformed, and then averaged to create a composite score for episodic memory and processing speed, respectively, at baseline and follow-up.

Automatic segmentation of white matter lesion burden

A FLAIR sequence was acquired with a total of 48 slices, slice thickness = 3 mm, TE = 120 ms, TR = 8000 ms, and field of view = 24 x 24 cm. WMLs were segmented with the lesion-growth algorithm (3), as implemented in the LST toolbox version 2.0.14 for SPM12. The algorithm first segmented the T1-weighted images into cerebrospinal fluid, grey matter, and white matter. This information was combined with the co-registered FLAIR intensities to calculate lesion-belief maps. These maps were thresholded (κ=0.3, defined by visual inspection) to obtain an initial binary lesion map, which was then grown along hyperintense neighboring voxels in the FLAIR image, and thresholded at 50% to yield a binary lesion map.

**References**

1. Greve DN, Svarer C, Fisher PM, Feng L, Hansen AE, Baare W, et al. Cortical surface-based analysis reduces bias and variance in kinetic modeling of brain PET data. Neuroimage. 2014;92:225-36.

2. Smith CT, Crawford JL, Dang LC, Seaman KL, San Juan MD, Vijay A, et al. Partial-volume correction increases estimated dopamine D2-like receptor binding potential and reduces adult age differences. J Cereb Blood Flow Metab. 2019;39(5):822-33.

3. Schmidt P, Gaser C, Arsic M, Buck D, Forschler A, Berthele A, et al. An automated tool for detection of FLAIR-hyperintense white-matter lesions in Multiple Sclerosis. Neuroimage. 2012;59(4):3774-83.

**Supplementary Table 1.**

White-matter lesion severity and cardiovascular risk factors in the intervention and control group. *p*>0.05 for all group comparisons.

|  | **Intervention** | **Active control** |
| --- | --- | --- |
| **White-matter lesions**  No lesions  Mild (Fazekas grade 1)  Moderate (Fazekas grade 2)  Severe (Fazekas grade 3) | 4 (13%)  18 (60%)  7 (23%)  1 (3%) | 1 (4%)  12 (43%)  14 (50%)  1 (3%) |
| Total amount (ml)  **Cardiovascular risk factors**  CVD risk profiles (%)  Systolic blood pressure  Diastolic blood pressure  BMI  Fat%  VO2 peak | 3.2±3.9  24.4±10.0  145.9±20.6  82.8±9.4  26.0±3.3  35.8±7.5  21.4±4.0 | 4.2±5.0  20.6±9.1  145.3±14.8  82.8±8.1  26.8±3.4  38.3±7.2  19.7±3.3 |

**
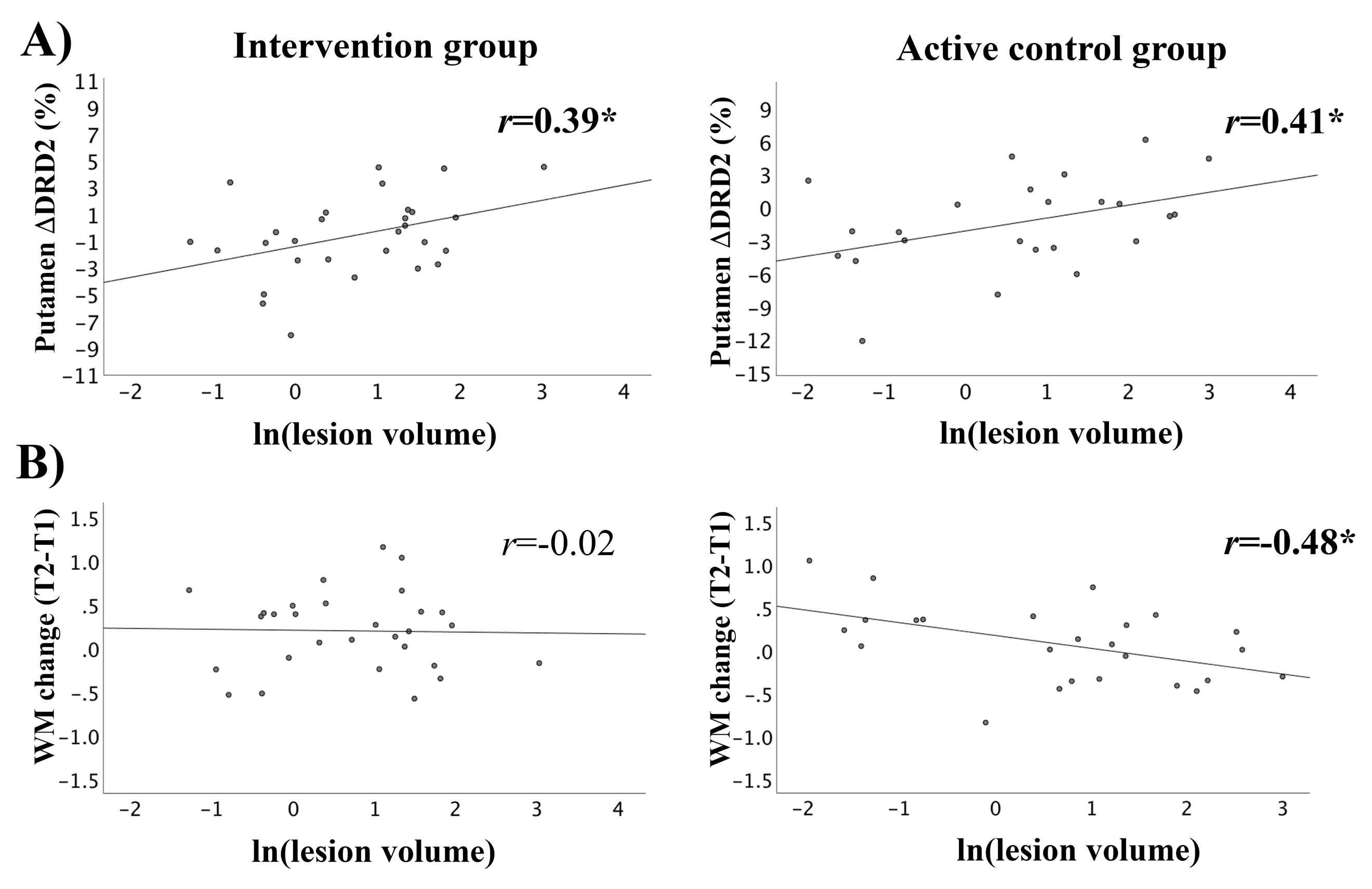
Supplementary Figure 1.** Zero-order correlations for white-matter lesion volume at baseline and putaminal D2-receptor change (A), and working memory change (B) for the intervention and active control groups, respectively. * *p*<0.05. Abbreviations: *r* (Pearson’s correlation coefficient), DRD2 (dopamine D2-receptor), T1 (timepoint 1 - baseline), T2 (timepoint 2 - follow-up); Δ (change).

**Supplementary Figure 2.** Links between lesion severity and putamen DRD2 availability in the intervention and active control group, respectively. * *p*<0.05. Abbreviations: DRD2 (dopamine D2-receptor), T1 (timepoint 1 - baseline), T2 (timepoint 2 - follow-up); Δ (change).


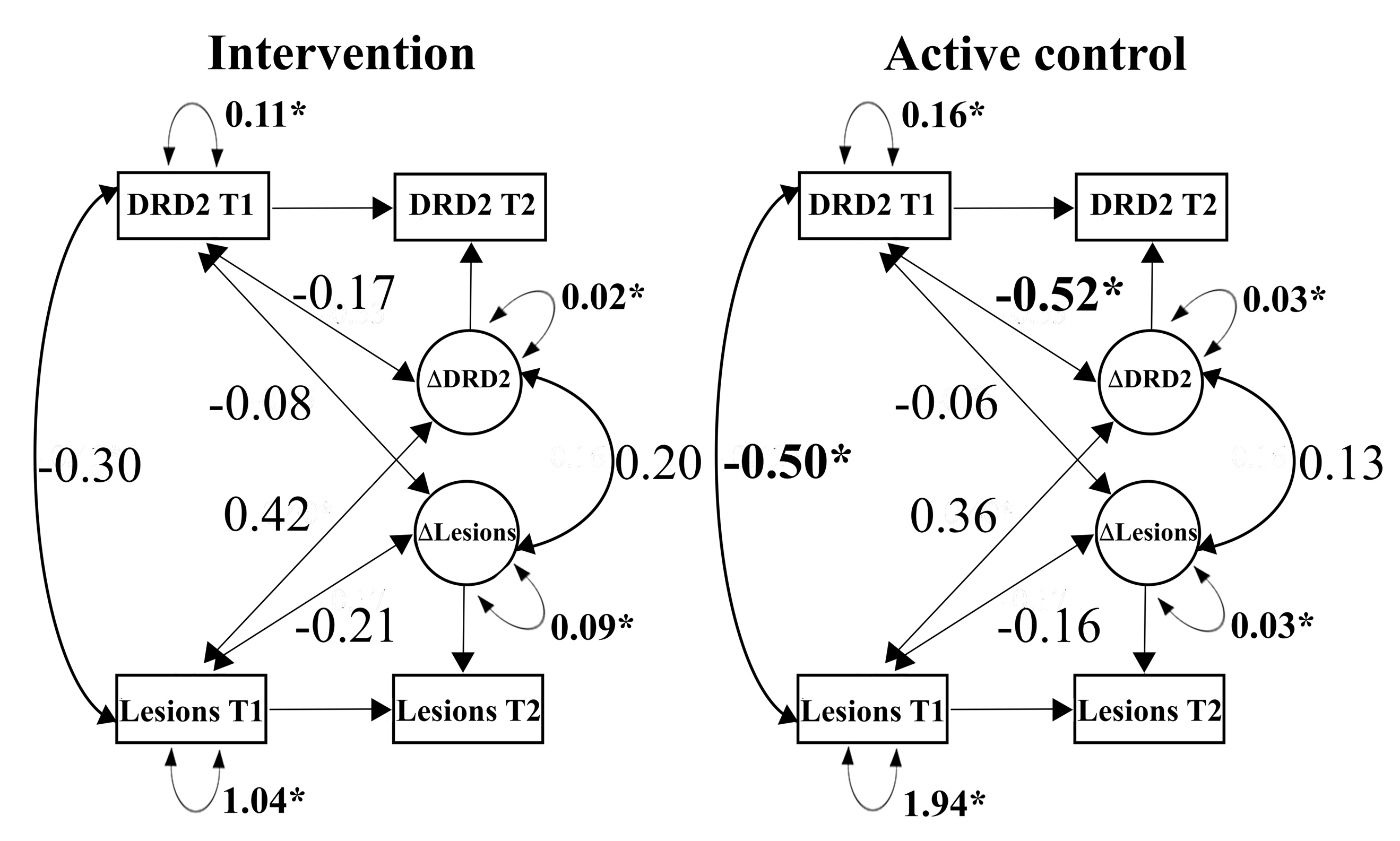


**Supplementary Figure 3.** Gains in perceptual speed are restricted to the group of individuals with milder lesion severity (A). No differences were observed between lesion severity groups for change in episodic memory (EM) performance (B). Performance is expressed as a Z-score. * *p*<0.05. Abbreviations: T1 (timepoint 1 - baseline), T2 (timepoint 2 - follow-up).
